# A propos d'une forme rare de la myosite ossifiante progressive de Munchmeyer

**DOI:** 10.11604/pamj.2015.20.416.4716

**Published:** 2015-04-28

**Authors:** Hicham Sator, Rachida Dafiri, Latifa Chat

**Affiliations:** 1 Hôpital des Enfants, Service de Radiologie, CHU Ibn Sina, Rabat, Maroc

**Keywords:** Congénital, enfant, myosite, ossification hétérotopique, radiologie, Congenital, child, myositis, heterotopic ossification, radiology

## Abstract

La myosite ossifiante progressive est une maladie génétique rare. On rapporte le cas d'un jeune garçon de 10 ans qui présentait de multiples tuméfactions douloureuses d'apparition spontanée et progressive au niveau du dos et des membres supérieurs. Ces tuméfactions étaient associées à une fébricule et un hallux valgus bilatéral. Les aspects radiologiques et tomodensitométriques étaient largement suffisants pour confirmer le diagnostic. Le traitement était purement médical. L’évolution était marquée par l'apparition d'autres ossifications des fascias et des muscles aboutissant à des raideurs articulaires très invalidantes. Les circonstances de découverte, les aspects épidémiologiques, étiopathogénique, diagnostique, évolutive et thérapeutique sont discutées à travers une revue de la littérature.

## Introduction

La myosite (ou fibrodysplasie) ossifiante progressive (MOP ou FOP), encore appelée maladie de MUNCHMEYER est une maladie génétique extrêmement rare à transmission autosomique dominante et à expression variable, caractérisée par des malformations congénitales des gros orteils et une ossification hétérotopique progressive des tissus musculaires et conjonctifs siégeant surtout au niveau de la région cervicale et dorsale, qui évolue par poussées et qui mène à des raideurs articulaires invalidantes. Les patients se voient comme enfermés dans un «deuxième squelette». Nous illustrons cette maladie par l'observation clinique d'un petit garçon de 10 ans afin d'en montrer les circonstances de découverte, les aspects épidémiologique, étiopathogénique, diagnostique, évolutive et thérapeutique.

## Patient et observation

**Figure 1:**
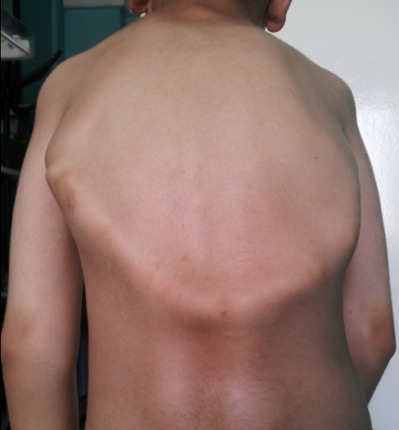
Aspect clinique du tronc montrant une cyphose dorsale, une raideur de tous le tronc, avec coulées osseuses sous cutanées, en arceau, en regard de grille costale postérieure reliant les deux humérus

**Figure 2:**
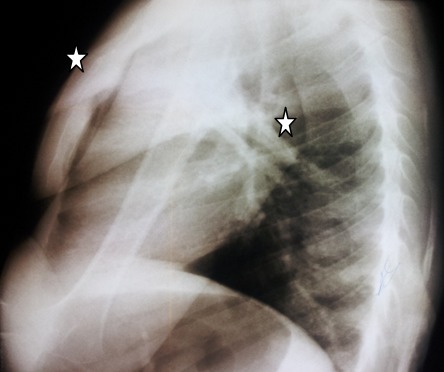
Radiographie thoracique profil: calcifications corticalisées irrégulières s’étendant en bande de rachis jusqu'au niveau des deux omoplates et des deux humérus

**Figure 3:**
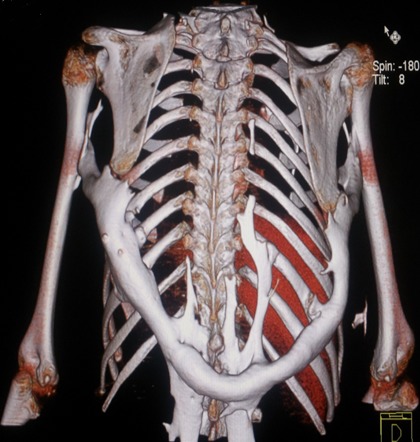
TDM thoracique avec reconstruction: importante ossification extrasquelletique engainant la face postérieure du tronc, dessinant un arceau a concavité supérieure et fusionnant avec les extrémités supérieures des humérus, associée à d'autres ossifications corticalisées en parasternale et en paravertébrale droit, et à une cyphose dorsale

**Figure 4:**
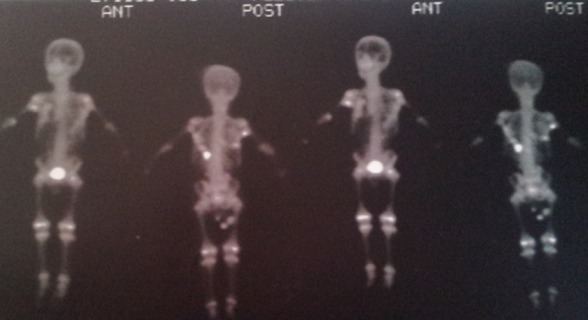
Scintigraphie osseuse a montré plusieurs foyers d'hyperfixation

M.M, jeune garçon de 10 ans sans antécédents familiaux particuliers, consultait pour ankylose des deux membres supérieurs à l'origine d'une impotence fonctionnelle invalidante. L'histoire de la maladie remontait à l’âge de quatre ans, par l'apparition spontanée, de façon bilatérale et asymétrique de tuméfactions douloureuses au niveau des muscles paravertébraux, augmentant progressivement de volume pour se fusionner et s’étendre jusqu'au niveau de l'extrémité supérieure des deux diaphyses humérales, limitant considérablement la mobilité de ces derniers et gênant sa vie quotidienne. L’évolution était marquée par l'apparition d'autres nodosités osseuses touchant les muscles parastérnales droit, ainsi que l'installation d'une cyphose dorsale d'aggravation progressive. A l'examen, il avait un bon développement psychomoteur et un discret retard staturo-pondéral. Il présentait une attitude guindé et une posture penché vers l'avant du fait de la cyphose dorsale et les nodules sous cutanés étaient immobiles, indolores, de consistance dure évoquant des excroissances osseuses, s’étendant en bande du gril costal postérieur à la face postéro-médiale des extrémités supérieures des deux bras latéralement et le long de rachis dorsale en haut plus marqué du coté gauche. Ces nodules étaient également présents au niveau parasternal droit (Figure 1). Les articulations scapulo-humérales avaient un secteur de mobilité nul dans toutes les directions. Les articulations des coudes, genoux et hanches étaient libres. L'examen des pieds a trouvé un hallux valgus bilatéral. Le bilan phosphocalcique était normal. La radiographie thoracique de face et de profil objectivait une ossification irrégulière s’étendant en bande de rachis jusqu'au niveau des deux omoplates et des deux humérus, sans lésions parenchymateuses pulmonaires (Figure 2). Le scanner thoracique avec reconstruction montrait la présence d'une importante ossification extrasquelletique engainant la face postérieure du tronc, et fusionnant avec les extrémités supérieures des humérus, associée à d'autres ossifications corticalisées en parasternale et en paravertébrale droit, et à une cyphose dorsale sans lésions parenchymateuses pulmonaires (Figure 3). La scintigraphie osseuse a montré plusieurs foyers d'hyperfixation (Figure 4). L'aspect de ces ossifications et leur association à un hallux valgus, nous ont permis de poser le diagnostic de myosite ossifiante progressive. Peur de favoriser leur extension, aucune biopsie ou intervention de libération de ces ossifications hétérotopiques n'a été indiquée.

## Discussion

La MOP, affection génétique extrêmement rare, touche en moyenne 1 personne sur 2 millions. Décrite pour la première fois en 1648 par PA TIN [1]. Cette affection est décrite essentiellement chez le jeune enfant et touche indifféremment les deux sexes: 41% des MOP sont dépistés avant l’âge de 2ans, 80% avant 10ans et 95% avant 15 ans [2]. Notre cas est en harmonie avec la littérature, les premiers symptômes s’étant manifestés à l’âge de 4 ans. Il ne semble pas que l'ethnie, la race, le sexe ou la géographie constituent des facteurs de prédisposition, cependant un facteur génétique est vraisemblable avec la survenue d'une mutation spontanée du gène ACVR1 portée sur le chromosome 4 de transmission autosomique dominante, codant pour un récepteur des protéines BMP4 impliquées dans la croissance et le modelage de l'os [3]. La symptomatologie de la MOP associe une fébricule à l'apparition de tuméfactions inflammatoires touchant les tissus conjonctifs et les muscles. Celles-ci vont s'ossifier et s’étendre progressivement à toutes les régions du corps. Ce processus affecte initialement les muscles du cou et les muscles paravertébraux avec une extension suivant un schéma proximo-distal et crânio-caudal [4]. Les atteintes vont alors toucher les épaules puis le rachis lombaire puis les hanches. Plus tard les articulations distales peuvent être atteintes vers la 3^ème^ décennie de la vie. Cependant selon Munchmeyer, les muscles qui ne s'insèrent pas sur le squelette par leurs deux extrémités sont épargnés. Ce qui explique le respect des muscles oculaires, du diaphragme, de la langue, du pharynx, du larynx et des muscles lisses [4].

Notre cas présente une grande similitude avec la littérature concernant les muscles cibles et la progression des lésions et tend à confirmer l'hypothèse de Munchmeyer. Par ailleurs, des malformations osseuses essentiellement présentes au niveau des pouces et des gros orteils sont mises en évidence dans 70à 100% des cas [4]. Elles se traduisent cliniquement par une microdactylie et un hallux valgus bilatéral. D'autres malformations plus rares et très variées sont décrites telles que des anomalies vertébrales, épiphysaires, rotuliennes, des épines calcanéennes, du col fémoral, des cinquièmes doigts ou encore des épaississements de la corticale interne du tibia. Dans notre cas, seul un hallux valgus bilatéral était observé. Des patients atteints de formes atypiques de FOP ont été décrits. Soit les signes classiques de la FOP sont présents, avec un ou plusieurs signes atypiques (par exemple: anémie aplastique intercurrente, craniopharyngiome, glaucome infantile ou retard de croissance) (FOP “plus”), soit l'un ou les deux signes cardinaux de la FOP présentent des variations majeures par exemple, des gros orteils normaux ou une réduction sévère des doigts (variantes de la FOP). En raison de l'ankylose des articulations costo-vertébrales et la déformation vertébrale, les patients finissent par développer une insuffisance respiratoire restrictive avec une atélectasie. La pneumonie et l'insuffisance cardiaque droite peuvent être mortelles. Les examens biologiques font partie des éléments diagnostiques de la MOP mais ils ont peu d'intérêt thérapeutique, évolutif et pronostic [5]. Le minimum d'examens biologiques à demander est relatif au métabolisme osseux dont le bilan phosphocalcique et le taux de PAL et celui relatif au processus inflammatoire dont la VS et la numération formule sanguine [5]. La radiologie conventionelle est la clé du diagnostic en montrant des images évocatrices telles qu'une calcification corticalisée ectopiques des muscles atteints et à un stade évolué, des ponts osseux entre les différentes parties du squelette avec un véritable squelette ectopique. Elle permet aussi de montrer des malformations osseuses congénitales typiques. Les autres moyens d'imagerie ne sont pas nécessaires pour le diagnostic, surtout à un stade évolué. Le scanner permet de mieux analyser les ossifications ainsi que leur étendue grâce aux reconstructions multiplannaires tels observés chez notre malade. L'IRM et la scintigraphie peuvent montrer des lésions au début non encore ossifiées [6]. Le diagnostic de la FOP est radio-clinique ne nécessitant pas de biopsie qui peut être le point de départ d'une ossification ectopique et peut induire en erreur, vu le caractère hétérogène des lésions [6].

Plusieurs diagnostics différentiels peuvent être évoqués surtout au début de la maladie et lorsqu'on n'examine pas les orteils et les doigts. Les métastases ossifiantes, les hématomes ossifiés, les tendinopathies calcifiantes, les maladies exostosantes, la myosite ossifiante circonscrite post-traumatique qui est généralement limitée à une seule localisation, délimitée, et douloureuse, qui survient à la suite de traumatismes. Les sarcomes des tissus mous peuvent être évoqués sur les résultats de la biopsie des lésions précoces [7, 8]. L'ostéodystrophie héréditaire d'ALBRIGHT est caractérisée par une ossification dans le muscle et le tissu conjonctif, mais plus marquée dans la graisse sous-cutanée et associée à une pseudohypoparathyroidie [9]. La spondylarthrite ankylosante pourra être évoquée devant une ankylose du rachis et des articulations sacro-iliaques [10]. L'histoire naturelle de cette affection est caractérisée par des poussées inflammatoires de 2 à 3 semaines, entrecoupées par des périodes de latence plus ou moins longues. L’évolution peut être émaillée par la survenue de plusieurs complications thromboemboliques, neurologiques, cutanées, infectieuses ou respiratoires. Son pronostic dépend surtout de la survenue d'une insuffisance respiratoire à un stade tardif.

À ce jour, aucun traitement n'a prouvé son efficacité. Cependant plusieurs moyens thérapeutiques sont proposés dans la prise en charge des MOP [1]. La kinésithérapie douce est proposée à visée antalgique lors des poussées. Le traitement médicamenteux fait appel aux corticoïdes, aux inhibiteurs des mastocytes, aux inhibiteurs de la cyclo-oxygénase 2, aux anti-inflammatoires non stéroïdiens aux amino-biphosphonates, aux antagonistes de BMP, aux agents anti-angiogéniques ou encore aux rétinoïdes avec des résultats encore aléatoires et insatisfaisants. Leur utilisation doit être mise en balance avec la sévérité potentielle de leurs effets secondaires. Il y'a peu de place pour la chirurgie d'autant plus que l'anesthésie de ces patients est difficile en raison de la rigidité rachidienne et de la fixation des mandibules [11, 12]. L'ablation chirurgicale de ces ossifications afin de mobiliser les articulations constitue un nouveau traumatisme qui favorise le développement d'ossifications hétérotopiques supplémentaires. Le seul but de la chirurgie est de corriger les attitudes vicieuses pour donner un enraidissement en une position la plus favorable possible. Devant les limites des traitements médical et chirurgical, la prévention devient très importante. Plusieurs mesures peuvent limiter l’évolution de la maladie: prévention des traumatismes, du blocage mandibulaire, médecine de réhabilitation et physiothérapie avec kinésithérapie respiratoire [13].

## Conclusion

Maladie extrêmement rare, la MOP doit être évoquée chez tout enfant présentant une ossification hétérotopique des parties molles, associée à des anomalies osseuses congénitales. Son diagnostic est radio-clinique. L’évolution est marquée par l'apparition de raideurs articulaires particulièrement invalidantes. Elle peut aussi mettre en jeu le pronostic vital de par les complications d'ordre général qu'elle occasionne. Aucun traitement préventif ou curatif efficace n'est disponible à ce jour. Néanmoins, plusieurs molécules sont en cours d'expérimentation
